# Fecal microbiota transplantation improves VPA-induced ASD mice by modulating the serotonergic and glutamatergic synapse signaling pathways

**DOI:** 10.1038/s41398-023-02307-7

**Published:** 2023-01-21

**Authors:** Jifeng Wang, Yuan Cao, Weiliang Hou, Dexi Bi, Fang Yin, Yaohui Gao, Dengfeng Huang, Yingying Li, Zhan Cao, Yinmei Yan, Jianhua Zhao, Dewu Kong, Xiaoqiong Lv, Linsheng Huang, Hui Zhong, Chunyan Wu, Qiyi Chen, Rong Yang, Qing Wei, Huanlong Qin

**Affiliations:** 1 Department of Pathology, Shanghai Tenth People’s Hospital Affiliated to Tongji University, 200072 Shanghai, China; 2 Intestinal Microenvironment Treatment Center, Shanghai Tenth People’s Hospital Affiliated to Tongji University, 200072 Shanghai, China; 3 Department of Pediatrics, Shanghai Tenth People’s Hospital Affiliated to Tongji University, 200072 Shanghai, China; 4 Shanghai Majorbio Bio-pharm Technology Co.,Ltd, 201210 Shanghai, China; 5 Intestinal Microenvironment Treatment Center, Shanghai Tenth People’s Hospital Affiliated to Tongji University, Department of Gastrointestinal Surgery, Shanghai Tenth People’s Hospital Affiliated to Tongji University, 200072 Shanghai, China

**Keywords:** Predictive markers, Autism spectrum disorders

## Abstract

Autism spectrum disorder (ASD) is a complex behavioral disorder diagnosed by social interaction difficulties, restricted verbal communication, and repetitive behaviors. Fecal microbiota transplantation (FMT) is a safe and efficient strategy to adjust gut microbiota dysbiosis and improve ASD-related behavioral symptoms, but its regulatory mechanism is unknown. The impact of the microbiota and its functions on ASD development is urgently being investigated to develop new therapeutic strategies for ASD. We reconstituted the gut microbiota of a valproic acid (VPA)-induced autism mouse model through FMT and found that ASD is in part driven by specific gut dysbiosis and metabolite changes that are involved in the signaling of serotonergic synapse and glutamatergic synapse pathways, which might be associated with behavioral changes. Further analysis of the microbiota showed a profound decrease in the genera *Bacteroides* and *Odoribacter*, both of which likely contributed to the regulation of serotonergic and glutamatergic synapse metabolism in mice. The engraftment of *Turicibacter* and *Alistipes* was also positively correlated with the improvement in behavior after FMT. Our results suggested that successful transfer of the gut microbiota from healthy donors to ASD mice was sufficient to improve ASD-related behaviors. Modulation of gut dysbiosis by FMT could be an effective approach to improve ASD-related behaviors in patients.

## Introduction

Autism spectrum disorders (ASDs) are complex neurobiological disorders characterized by altered social interaction and repetitive and stereotyped behavior [1]. Recent epidemiological studies have reported a dramatic increase in the prevalence of ASD, with one morbidity in every 45 children [2]. The pathogenesis of ASD involves a multifaceted interaction between genetic and environmental factors, and no efficient diagnostic test or cure has been available for autism until now [3]. Recent studies have shown that ASD children have an altered gut microbiome composition compared with that of normal children [4]. The obstipation, chronic diarrhea, and abnormal distension of the gastrointestinal (GI) tract occurring in ASD children might be linked to GI microbiota dysbiosis [5, 6]. With the increasing knowledge of the gut microbiome impacting ASD, gut microbiota modulation to restore gut microbial homeostasis has become a potential strategy for ASD treatment. In particular, fecal microbiota transplantation (FMT) has been proposed to be a safe and efficient strategy to adjust GI microbiota symbiosis and improve behavior symptoms for ASD children [7, 8]. Although the gut microbiome emerges as an integral player in gut-brain communication [9–11], there is still much that remains unknown about the brain-gut-microbiome axis in ASD, especially the effect of specific microbiota and their function on ASD development and/or the improvement in ASD-associated behaviors.

It is not clear which components of the FMT material, that is, the microbes or their products, mediate behavioral changes. Studies in animal models have shown that gut microbes can modulate central nervous system-related behaviors [12–14]. An imbalance in the gut microbial populations has been associated with ASD mouse models [15, 16]. Exposure to valproic acid (VPA) during pregnancy has been demonstrated to increase the risk of autism in children [17, 18]. Animal studies have shown that rodents prenatally exposed to VPA display behavioral phenotypes characteristic of the human condition, which represents a robust model of autism exhibiting face, constructive and predictive validity. This model has been used extensively to investigate the molecular pathways linked to autistic behavior and to test potential therapeutics for autism [19–21].

In this study, we aimed to investigate the reconstitution mechanism of the gut microbiota and to explore the therapeutic potential for ASD by restoring the healthy microbiome by FMT. The fecal microbiota from ASD children and the screened FMT donors (TD) was transplanted into a VPA mouse model. After FMT, we performed 16 S rRNA gene sequencing of mouse fecal samples to identify the difference in the gut microbial communities. The untargeted metabolomic analysis of mouse serum samples was carried out to explore the perturbation of metabolic pathways for ASD behaviors. In addition, we conducted mRNA sequencing analysis of colon and brain tissues from a mouse model to identify significantly enriched key signaling pathways and preliminarily explored the possible underlying molecular mechanisms. This preclinical study provides novel insights into ASD research and potential therapeutic prospects for ASD children.

## Materials and methods

### Animals

Specific pathogen-free (SPF) C57BL/6 J mice (Shanghai Jihui Laboratory Animal Care Co.,Ltd.) were mated and examined for the presence of a vaginal plug, which was used to define embryonic day 0 (E0). On E12.5, all pregnant mice were intraperitoneally injected with 500 mg/kg of valproic acid (VPA) (Sigma Aldrich, MO, USA) in 0.9% saline, while control animals were instead injected with an equivalent volume of saline solution. On postnatal day 14, maternal and offspring were administered to antibiotic mix containing ampicillin (1 g/L), vancomycin (0.5 g/L), metronidazole (1 g/L) and neomycin (1 g/L) in drinking water. On postnatal day 21, the male offspring were separated and treated with ABX for another week, then randomly allocated to different groups and housed (4 per cage) for the subsequent ASD-related experiments. Animals were housed in cages under standard laboratory conditions (12 h dark/light cycles) with free food and water access. All animal studies were conducted in accordance with a protocol approved by the Institutional Animal Care and Use Committee of Tongji University.

### FMT and experimental grouping

FMT was performed according to a modified method described previously. We selected four randomly patients with ASD (Supplementary table 1) and two screened healthy donors (TD) which has been used in another clinical trial with promising clinical application (ChiCTR2100043906). The studies involving human participants were reviewed and approved by Ethics Committee of Shanghai 10th People’s Hospital. The patients/participants provided their written informed consent to participate in this study. The fecal sample collection was conducted with the approval from the Ethics Committee of the Tenth People’s Hospital Affiliated to Tongji University and in accordance with the ethical principles for the medical research outlined in the Declaration of Helsinki 1964 as modified by the subsequent revisions.

The fecal supernatants of four ASD donors and two TD donors were used as a single source for mice, respectively. Following antibiotic treatment, the recipient mice were orally inoculated every other day for 21 days with prepared fecal contents. The control group of male mice was gavaged as described previously, but the donor supernatant was replaced with phosphate-buffered saline (PBS). Mice harboring microbiota from patients with ASD and healthy donors, as well as PBS, were referred to as VPA_FMT_ASD, VPA_FMT_TD and VPA, respectively.

### Behavioral studies

After 3 weeks FMT, mice underwent social interaction, open field, elevated plus maze tests. On the day of testing, animals were brought in sterile filtered cages to an adjacent testing room and allowed to rest for 1 h before testing. Test chambers were cleaned first with disinfectant and then with 70% ethanol and water after each animal.

### The three-chamber social interaction test

The three-chamber social interaction test consisted of three equally sized rooms (20 cm × 45 cm each) divided by clear Plexiglas, and with an access door between each compartment. Test animals were sex-and strain-matched with unfamiliar mice with which they had not had any prior contact. At the start of the test, a selected mouse was placed in the empty central chamber, with the selected unfamiliar mouse being placed in a wire cage in the one chamber, and an empty wire cage (novel object) being placed in another compartment. Following a 5 min acclimation period, sociability tests were conducted for 10 min. For social novelty preference tests, a different novel animal was positioned within the wire cage in another chamber, and the study subject was returned to the empty central chamber. Animal movements during the following 10 min were then monitored, with animals having free access to all three chambers.

### Open-field test

Locomotor activity was monitored for 10 min in an open field, a white Plexiglas box (50 cm × 50 cm) with its floor divided into 9 squares. Each animal was gently placed in the centre of the box, and activity was scored. The following parameters were considered: the time spent in the centre, the total distance moved, and the mean velocity during the 10-min test. An increase of time spent in the centre or a decrease of the latency to enter the centre are indicators of anxiolytic activity and vice versa.

### Elevated-plus maze (EPM)

The EPM consists of 2 opposing open arms and 2 opposing closed arms of the same size (45 cm × 10 cm) with 10 cm high walls connected by a central platform (10 cm × 10 cm) and elevated 80 cm above the floor. Mice were positioned in the central platform facing a closed arm and the number of entries into, time spent on each arm, and central platform was measured. The maze was systematically cleaned to remove olfactory cues, after each animal was tested. The shorter the time spent in open arms and central platform, the higher the anxiety was and vice versa. Mean velocity and total distance moved was also measured and examined for every experimental group.

### Behavioral analysis

The behavioral data from the open field test were analyzed by using either repeated measures analysis of variance (ANOVA; phenotype and time as main factors) or factorial ANOVA when appropriate. The behavioral data from elevated-plus maze tests were analyzed by using one-way ANOVA. All post hoc comparisons were made with Bonferroni/Dunn test in the presence of significant ANOVA effects. The threshold for statistical significance was set as *P* ≤ 0.05.

### Tissue and sample collection

After the behavioral experiments, the mice were sacrificed and blood, brain and colon tissue were collected for further study.

### RT-PCR analysis

Total colon RNA was extracted with the Rneasy Mini kit (Qiagen, Hilden, Germany). Two micrograms of total RNA was reverse transcribed using the Transcriptor First Strand cDNA Synthesis Kit (Roche Applied Science). Real-time monitoring of PCR amplification of cDNA was performed using DNA primers and the ABI PRISM 7500 HT Sequence Detection System (Applied Biosystems) with SYBR PCR Master Mix (Applied Biosystems). Target gene expression was normalized to glyceraldehyde-3-phosphate dehydrogenase (GAPDH) levels in the respective samples as an internal standard, and the comparative cycle threshold (Ct) method was used to calculate relative amount of target mRNAs. Oligonucleotide primers used for PCR amplification of human GAPDH, Shank1,Shank3 and Maml3 were as follows: for GAPDH, sense (5'-TCCTGTTCGACAGTCAGCCGCA-3') and anti-sense (5'-ACCAGGCGCCCAATACGACCA-3'); for Shank1, sense (5'-ATGGCTGAA GAGGGCTGAAAT-3') and anti-sense (5'- GCACTGTGTCTTTCCTCGTC-3'); for Shank3, sense (5'-GGACCCTGCTAAGAAGTCACC-3') and anti-sense (5'- CAGCATGTGGACTGCGAGAG-3'); for Gadd45, sense (5'-GAGGACACATG GAAGGACCC-3') and anti-sense (5'-TCACTCGGGAAGGGTGATG-3'); for Maml3, sense (5'-ACGTATGGCAACTTCTCCGTT-3') and anti-sense (5'- TCAGAGTCGTGTACATCCCA-3').

### Immunohistochemistry (IHC)

Brain and colon tissues were fixed, embedded in paraffin, and processed on slides for Immunohistochemistry (IHC). Histopathological staging was performed by an experienced pathologist. Primary monoclonal antibodies used to perform immunostaining were: anti-c-Fos (ab214672, 1:1000, Abcam), anti-TH (ab6211, 1:500, Abcam).

### 16S rRNA gene sequencing

Total genomic DNA was extracted from the fecal DNA kit (catalog number DP712; Tiangen, China) according to the manufacturer’s protocol. DNA concentration and purity were monitored on 2% agarose gels. According to the concentration, DNA was diluted to 1 ng/ml using sterile water. The V4 regions of the bacterial 16 S rRNA gene were amplified with the barcoded primers 341 F (5'-CCTACGGGRSGCAGCAG-3') and 806 R (5'-GGACTACVVGGGTATCTAATC-3') by an ABI GeneAmp® 9700 PCR thermocycler (ABI, CA, USA). After preparation of library, these tags were sequenced on NovaSeq PE250 platform (Illumina, San Diego,USA) according to the standard protocols by Majorbio Bio-Pharm Technology Co. Ltd. (Shanghai, China) for paired-end reads of 250 bp. After the sequencing, all reads were sorted, screened, and filtered to ensure quality and length. The denoised reads were dereplicated to a unique sequence and were then sorted by abundance and subjected to operational taxonomic units(OTU) clustering at 97% similarity. The representative sequences were classified into organ-isms by a naïve Bayesian model using RDP classifier (V.2.2) based on Greengenes (V.gg_13_5) (http:// greengenes. secondgenome. com). The abundance statistics of each taxonomy and phylogenetic tree was construction in a Perl script and visualized using Scalable Vector Graphics. Principal coordinate analysis (PCoA) of weighted unifrac distances was calculated and plotted in R.

### Metabolome analysis

The non-targeted metabolomics approach was employed for serum metabolomics. In brief, 50 mg of serum samples was precisely weighed and added to 400 μL extraction solution (methanol: water = 4:1).The obtained solution was vortexed for 30 s followed by low-temperature sonication for 30 min of extraction (5 °C, 40 KHz). The extracted samples were left to stand for 30 min followed by 15 min of centrifugation (13,000 g, 4 °C) and the supernatant was collected. Lastly, differential metabolites (DMs) of the supernatant were analyzed using liquid chromatography-mass spectrometry (LC-MS).

### Transcriptome analysis

The transcriptome analysis was performed as previously reported (Li et al., 2019; Lin et al., 2020). In brief, the total RNA of the cortex and colon were extracted by the TRIzol reagent kit (Invitrogen). RNA sample quality was then determined using 2100 Bioanalyser (Agilent) and ND-2000 (NanoDrop Technologies). Later, RNA-seq transcriptome librariy was prepared following TruSeqTM RNA sample preparation Kitfrom Illumina (San Diego, CA) using 1 μg of total RNA. Lastly, samples were loaded on Illumina Nova seq 6000 to be sequenced. Detailed information on transcriptome analysis is presented in the Supplementary Information. To validate the reliability of changes in tissue transcript levels induced by FMT exposure, 11 differentially expressed genes (DEGs) at the transcript level (i.e., gene) were selected for quantitative real-time polymerase chain reaction (qRT-PCR). Detailed information on qRT-PCR analysis is presented in the.

### Data analysis

In this study, analysis of all omics data was performed using a free online platform of Majorbio Cloud Platform, https://cloud.majorbio. com (Shanghai Majorbio Bio-pharm Technology Co., Ltd). The normality of non-omics data (e.g. biomarkers and histology) was assessed using the Kolmogorov-Smirnov test. To discriminate significant differences relative to the control group, one-way analysis of variance (ANOVA) was then performed followed by the Duncan test using SPSS 22.0 software. *p* < 0.05 was considered to be a significant difference between treatments and the control group.

## Results

### FMT from screened donors alleviated core symptoms of ASD in mice

**Fig. 1:**
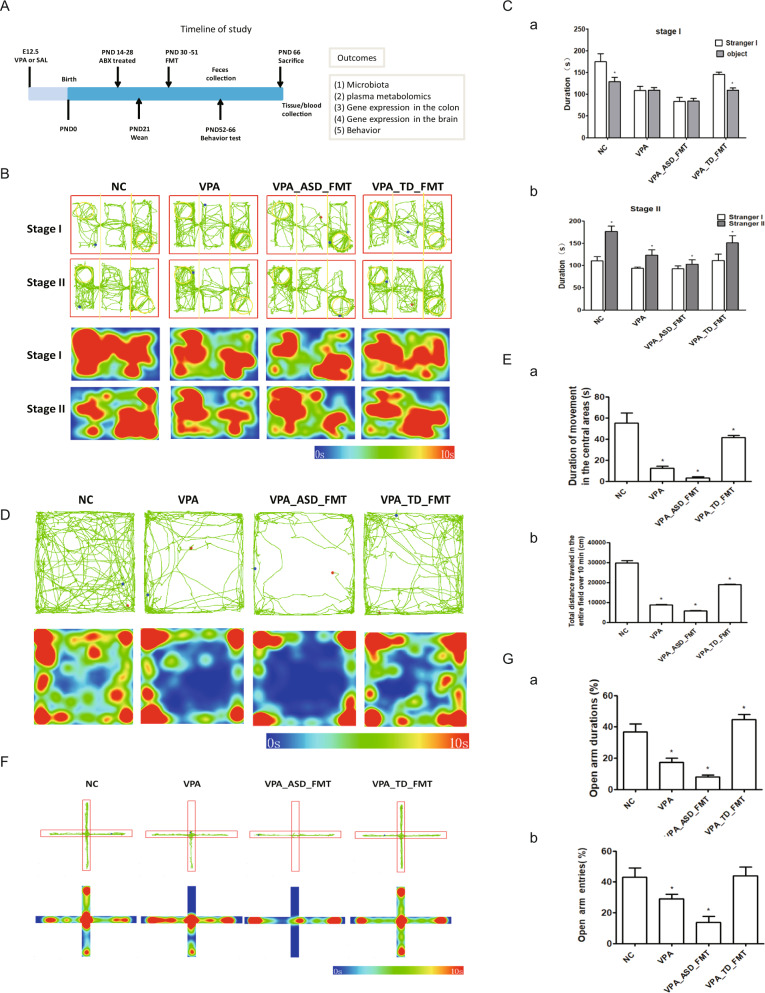
Behavioral tests on VPA-treated mice colonized with fecal samples from control (TD) donors and donors with autism spectrum disorder (ASD). **A** Experimental timeline of the animal experiment. E12.5, embryonic day 12.5; VPA, valproic acid; PND, postnatal day. **B**, **C** Three-chamber sociability test: After a 10-min habituation to a three-chambered box, an empty cup and a cup containing stranger 1 were introduced in the side chambers for a 10-min sociability session. Thereafter, stranger 2 was added under the empty cup for a 10-min social novelty session. **B** Representative tracks (top two panels) and heatmap of the overall activity (bottom two panels) of mice in the three-chamber sociability test. **C** (**a**) Time spent in each side chamber containing the strange 1 mouse or empty wire cage. (**b**) Time spent in each side chamber containing a familiar mouse (strange 1) or a strange 2 mouse 10 min after the first exposure. **D**, **E** Open-field test: (**D**) Representative tracks (upper panel) and heatmap of the overall activity (lower panel) of mice in the open field chamber over 10 min; (**E**) (**a**) Comparison of times spent in the center zone among groups. (**b**) Total distance traveled in the entire field over 10 min of groups. **F**, **G** Elevated plus maze test: (**F**) Representative tracks (upper panel) and heatmap of the overall activity (lower panel) of mice in the elevated plus maze; (**G**) (**a**) Percentages of time spent in the open arms (%) were measured. (**b**) Percentages of open arm entries (%) were measured. *P, 0.05; VPA_TD_FMT, mice transplanted with the fecal microflora of the TD donors; VPA_ASD_FMT mice transplanted with the fecal microflora of donors with ASD.

To investigate the contribution of the gut microbiome composition to the treatment of ASD, we examined the effect of fecal microbiota transplantation (FMT) on a valproic acid (VPA)-induced autism mouse model (Fig. 1A). VPA-mice were first antibiotic-treated for 1 week and then randomly separated into three groups (7 animals in each group). A pooled fecal sample from four randomly selected ASD donors or two screened healthy donors (TD) that were used in another clinical trial with promising clinical application (clinically effective FMT samples) or vehicle solution (saline) was colonized in ASD mice by FMT, and the groups were named the VPA_ASD_FMT group, VPA_TD_FMT group, and VPA group, respectively. After FMT, behavior tests were carried out using a three-chamber model, open-field model and elevated plus maze model. Then, the mice were sacrificed, and tissue samples (feces, blood, colon, and brain) were obtained to measure the gut microbiota, metabolome and gene expression.

In the three-chamber sociability test (first stage), the VPA_ASD_FMT group displayed a significantly greater preference for the novel object chamber and a lower preference for the strange mouse chamber (Fig. 1B, C). In contrast, the VPA_TD_FMT group spent significantly longer time in the strange mouse chamber than in the novel object chamber, while the VPA group mice showed no different attitude toward the two stimuli. Similarly, in the second stage (social novelty test), the VPA_ASD_FMT group displayed a significantly greater preference for the familiar-strange-mouse chamber and a lesser preference for the new-strange-mouse chamber. The VPA_TD_FMT group displayed a significantly greater preference for the new-strange-mouse chamber and a lower preference for the familiar-strange-mouse chamber (*P* < 0.05) (Fig. 1C), indicating that modification of the gut microbiota by FMT with TD samples restores recognition behavior and social novelty responses.

During the open-field test, the movement time of the VPA_ASD_FMT group was significantly shorter in the center zone than that of the VPA group (*P* < 0.05) (Fig. 1D, E). Interestingly, the percentage of center time in the VPA_TD_FMT group was much longer than that of VPA group mice (*P* < 0.05). These results suggested that FMT with ASD samples aggravated the anxiety level of mice, and mice were less likely to explore the unknown area due to more anxiety and fear. While FMT with TD samples alleviated the anxiety level of mice, mice were more likely to explore the unknown area due to less anxiety and fear.

Furthermore, in the elevated plus maze test, FMT with ASD samples was associated with decreased time spent in the open arms relative to that of control VPA group mice, while FMT with TD samples was associated with increased time spent in the open arms compared with that of VPA and VPA_ASD_FMT group mice. The total distance traveled was comparable in these groups (Fig. 1F, G), indicating that the VPA_TD_FMT group had higher locomotion and lower anxiety in the elevated plus-maze test. Taken together, these behavior tests suggest that the gut microbiota of donors could alleviate core symptoms of ASD in mice.

### The microbiota of mice acquired a similar structure following FMT from donors

**Fig. 2:**
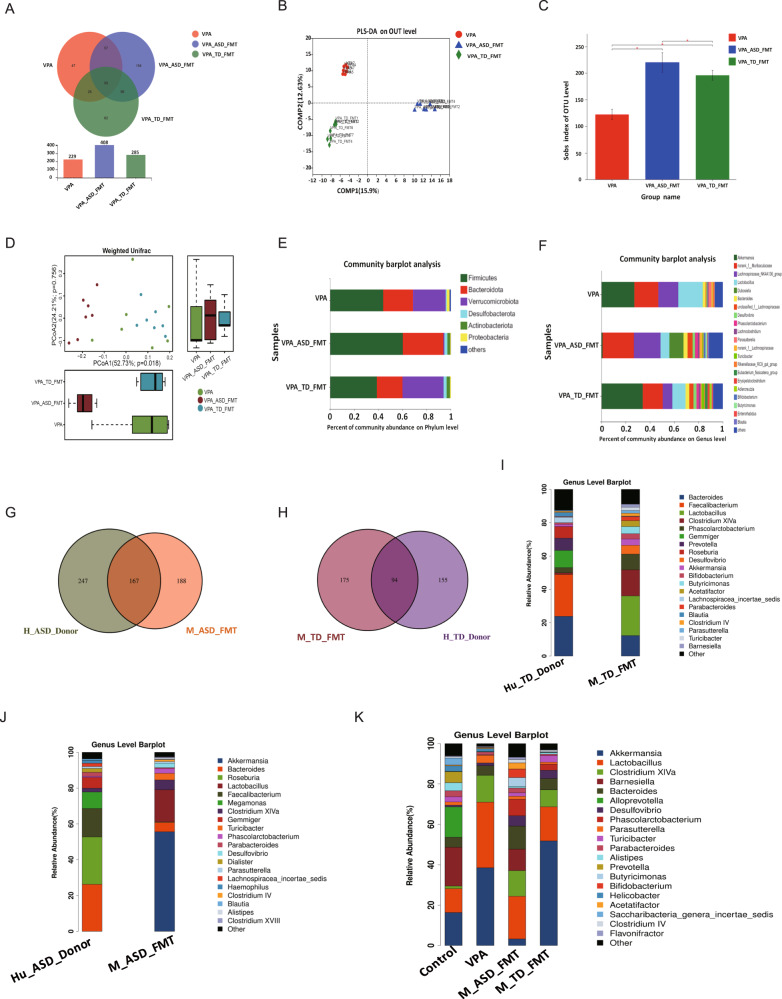
Changes in the gut microbiota composition in VPA-treated mice after FMT. **A** Venn diagram depicting the number of bacterial taxa that were unique and shared between the VPA, VPA_ASD_FMT and VPA_TD_FMT groups; (**B**) PLS-DA score plot based on OTUs of the microbial community in the three groups. Smaller distances between two points indicate greater similarity in microbial community structure between the two samples. PLS-DA: partial least squares discriminant analysis; (**C**) Alpha-diversity of the gut microbiota (sob index) in the three groups; (**D**) Beta-diversity of the gut microbiota in the three groups; (**E**) Relative abundances of bacterial taxa at the phylum level; (**F**) Relative abundances of bacterial taxa at the genus level. Only phyla with an average relative abundance >1% and genera >5% are shown. **G** Venn diagram depicting the number of bacterial taxa that were unique and shared between the Human_ASD_donor and VPA_ASD_FMT groups. **H** Venn diagram depicting the number of bacterial taxa that were unique and shared between the Human_TD_donor and VPA_D_FMT groups. **I** Relative abundances of bacterial taxa in the Human_ASD_donor and VPA_ASD_FMT groups at the genus level. **J** Relative abundances of bacterial taxa in the Human_TD_donor and VPA_TD_FMT groups at the genus level. **K** Relative abundances of bacterial taxa in the four groups of mice at the genus level.

We then compared the gut microbial composition using 16 S ribosomal RNA gene sequencing in fecal samples to detect species differential abundance in these groups. Venn (Fig. 2A) and partial least squares discriminant analyses (Fig. 2B) revealed conspicuous differences in microbial community structures among the VPA, VPA_ASD_FMT and VPA_TD_FMT groups. Statistically significant differences were found in the alpha-diversity (Fig. 2C) and beta-diversity (Fig. 2D) among the three groups. This finding shows that microbial communities in the VPA, VPA_ASD_FMT and VPA_TD_FMT groups formed distinct characteristics. Abundance changes in the gut microbiota at the taxonomic level were also evaluated. The dominant microbiota compositions of the VPA, VPA_ASD_FMT and VPA_TD_FMT groups at the phylum level are shown in Fig. 2E, F. We found that VPA-treated ASD mice showed a higher F/B ratio than control mice. After FMT, the recipient mice had a microbiota structure similar to that of their donors, showing the difference in the microbiome profiles for the TD donors and ASD donors.

**Fig. 3:**
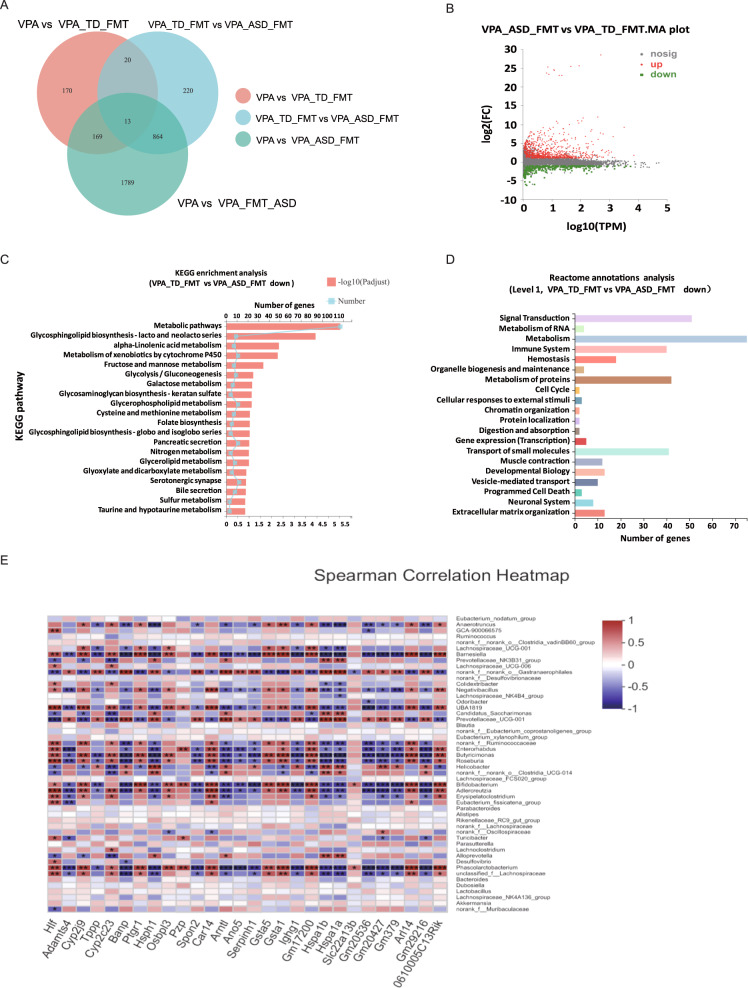
Gene expression profiling analysis of colon tissues from VPA-treated mice after FMT treatment. **A** Venn diagram showing the total number of expressed genes in each group in the large circles, and the number of genes expressed in both groups are shown in the overlapping portion of the circles. **B** Volcano map showing the significantly differentially expressed genes represented by red dots (upregulated) and green dots (downregulated) for the VPA_TD_FMT group and the VPA_ASD_FMT group. **C** KEGG enrichment analysis showing the top 20 signaling pathways affected by DEGs between the VPA_TD_FMT group and the VPA_ASD_FMT group. **D** Reactome annotation analysis of the DEGs between the VPA_TD_FMT group and the VPA_ASD_FMT group. Twenty statistically significant pathways are listed. **E** Correlation between the changes in the relative abundances of individual genera and DEGs involved in the serotonergic synapse pathway and glutamatergic synapse pathway in the colon tissue of the VPA_TD_FMT group and the VPA_ASD_FMT group. *P*-values were corrected for multiple testing using the Benjamini‒Hochberg false discovery rate. *p < 0.05, **p < 0.01.

The Venn diagram plot of the gut microbiota indicated the distribution of OTUs shared by the human donor and mice after 3 weeks of FMT (Fig. 2G, H). Then, we analyzed the OTUs shared by the human donors that were absent from the VPA group. Indeed, the OTUs corresponded to those successfully transplanted from humans to mice following FMT. We analyzed the relative abundances of human donors and mice to identify specific bacteria that were transferred to mice following FMT (Fig. 2I, J, K). In detail, the differential abundance analysis (on the OTU table at the genus level of taxonomic assignment) indicated behavioral changes associated with the taxonomic profile. The VPA_ASD_FMT group had higher relative abundances of the genera *Bacteroides* and *Odoribacter*, among others. The VPA_TD_FMT group had higher abundances of genera such as *Akkermansia* and *Erysipelatoclostridium* (FDR *p*-value < 0.05) (Fig. 3A). In particular, we found that the relative abundance increases of *Turicibacter* and *_Alistipes* were also significantly positively correlated with the improvement in behavior after FMT, which is in accordance with the metagenomic analysis of our ongoing clinical research project on ASD treatment with FMT (data not shown).

### Activation of the serotonergic synapse pathway and glutamatergic metabolism in the colon of mice via FMT

We employed colon RNA sequencing from the VPA, VPA_TD_FMT and VPA_ASD_FMT groups to characterize the effect of FMT on colon tissue gene expression in the host. Venn analysis revealed transcriptional changes in the colon tissue (Fig. 3A). Differentially expressed genes (DEGs) screened in the VPA_TD_FMT group versus the VPA_ASD_FMT group are presented as a volcano plot graph (Fig. 3B). Kyoto Encyclopedia of Genes and Genomes (KEGG) enrichment analysis indicated that DEGs were significantly concentrated in metabolic pathway, glycosphingolipid biosynthesis, alpha-linolenic acid metabolism, serotonergic synapse pathway and glutamatergic metabolism, which are involved in brain disorders, including ASD (Fig. 3C). In view of the pathways in the Reactome annotation analysis (Fig. 3D), 8 DEGs were concentrated in the neuronal system, which is involved in the pathways of GABA synthesis, release, reuptake and degradation, GABA and GABA B receptor activation, the dopamine and serotonin neurotransmitter release cycle, presynaptic and postsynaptic nicotinic acetylcholine receptors and neurotransmitter uptake and metabolism in cells. We also found 7 DEGs involved in the recycling of glutamate, including glutamate and glutamine metabolism (2 genes), astrocytic glutamate-glutamine uptake and metabolism (1 gene), the glutamate neurotransmitter release cycle (1 gene), glutathione conjugation (2 genes), and activation of kainate receptors upon glutamate binding (1 gene).

According to the aforementioned findings, we attempted to identify microbial taxa that likely drive the expression of specific host genes after FMT. Correlation analysis illustrated that the abundant genera of the VPA_TD_FMT group, such as *Bacteroides*, *Odoribacter, Phascolarctobacterium, Adlercreutzia, Bifidobacterium, Roseburia* and *Anaerotruncus*, were associated with DEGs (Fig. 3E). These results indicate a potential transcriptional regulation mechanism by gut microbiomes after FMT treatment.

### FMT activated glutamatergic synapse metabolism pathways in the blood of mice

**Fig. 4:**
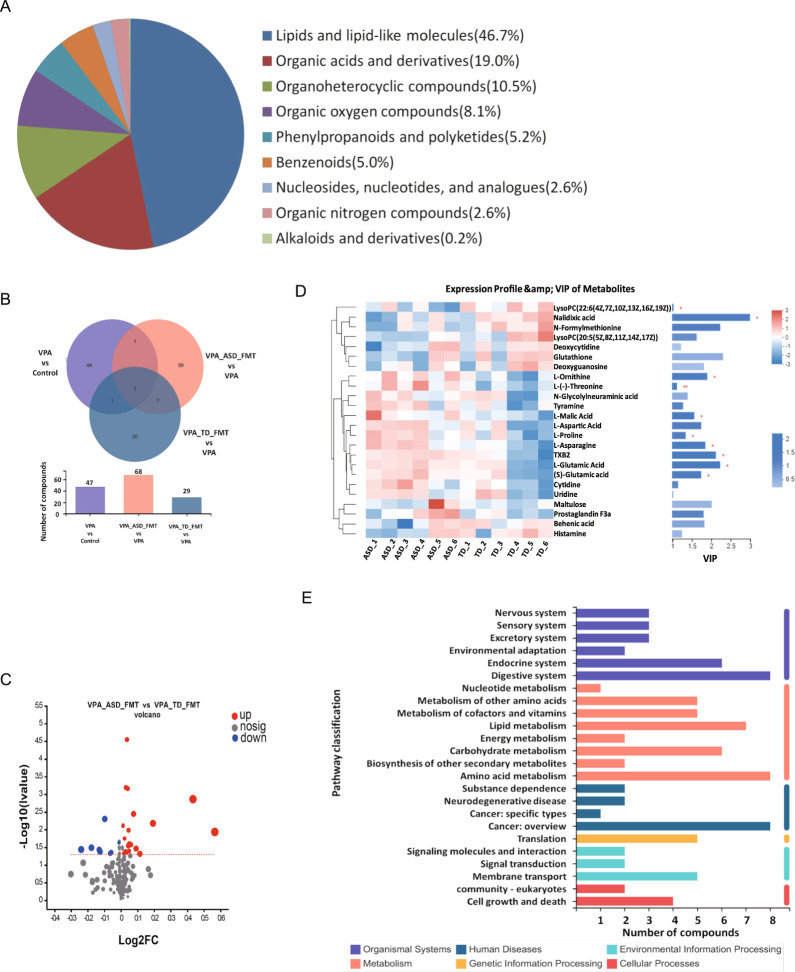

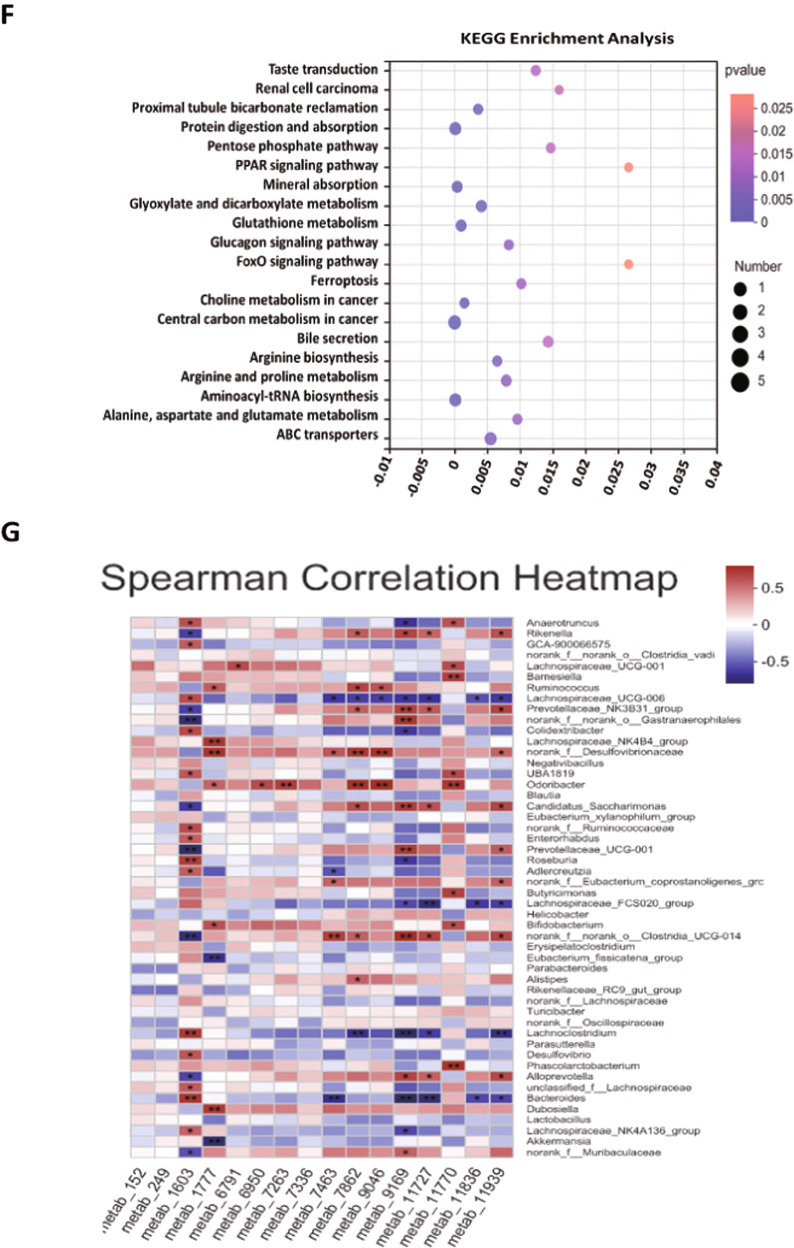
Effects of FMT on the metabolic differences in the serum samples of VPA-treated mice. **A** HMDB compound classification of serum metabolites between the VPA_TD_FMT group and the VPA_ASD_FMT group; (**B**) Venn diagram showing the total number of metabolites in each group in the large circles, and the number of metabolites in both groups is shown in the overlapping portion of the circles; (**C**) Volcano plot of the differential metabolites of serum in the VPA_TD_FMT group and the VPA_ASD_FMT group; (**D**) Heatmap showing the abundances of the top 24 differential metabolites based on VIP scores of the VPA_TD_FMT group and the VPA_ASD_FMT group; (**E**) Serum KEGG pathways between the VPA_TD_FMT group and the VPA_ASD_FMT group; (**F**) KEGG enrichment pathways for serum metabolites in the VPA_TD_FMT group and the VPA_ASD_FMT group; (**G**) Correlation between the changes in serum metabolites and changes in genus abundance. The heatmap shows the Spearman correlation coefficient between the changes in serum metabolite concentrations and changes in the relative abundances of individual genera. The intensity of the colors represents the degree of association between the changes in the concentrations of serum metabolites and changes in the relative abundances of individual genera as measured by Spearman’s correlations. *P*-values were corrected for multiple testing using the Benjamini‒Hochberg false discovery rate. *p < 0.05.

Then, we performed untargeted metabolomics analyses of serum samples collected from the VPA, VPA_ASD_FMT and VPA_TD_FMT groups to discover different metabolites (DMs) and metabolic pathways of the intestinal microbiome that might be involved in ASD. The serum samples from the VPA, VPA_ASD_FMT and VPA_TD_FMT groups were largely separated according to principal component analysis (PCA), as shown in Fig. 4A. Chemical profiling of serum metabolomes revealed diverse chemical groups belonging primarily to amino acid, peptide, and analog subclasses; glycerophosphocholine subclasses; fatty acid and conjugate subclasses; carbohydrate and carbohydrate conjugate subclasses; as well as the sesquiterpenoid subclass (HMDB annotation). We identified 123 differentially expressed metabolites in serum samples among the VPA, TD-FMT and ASD-FMT groups (Fig. 4B). Differential metabolites with fold changes ≥ 1.5 and ≤2/3 (FDRb0.05) were screened in the VPA_TD_FMT group versus the VPA_ASD_FMT group and are presented as a volcano plot graph (Fig. 4C). The different metabolites were further screened using variable importance in projection (VIP) (Fig. 4D). The differential metabolites were entered into the KEGG database to construct and analyze metabolic pathways. Interestingly, the differentially expressed metabolites in the serum samples of VPA_ASD_FMT and VPA_TD_FMT group mice were significantly enriched in specific metabolic pathways, including L-glutamic acid, (S)-glutamic acid, glutathione, oxidized L-proline, and L-asparagine (Fig. 4E, F), which were enriched in glutamatergic synapse metabolism and GABAergic synapse pathways.

On the basis of the differences between the gut microbes and metabolites among the different treatment groups, the functional correction between the changes in the gut microbiome and metabolite profiles was investigated by calculating Spearman’s correlation coefficient (Fig. 4G). A significant correlation was identified between the changes in the gut microbiome and metabolite profiles. The results revealed that the relative abundance of several bacterial families significantly correlated with the levels of multiple metabolites in the host serum, such as *Bacteroides, Odoribacter, Lachnospiraceae_FCS020_group, Rikenella*, and *Lachnospiraceae_UCG-006*. Interestingly, some metabolite levels were highly correlated with the abundance of some microbes, including glutathione (oxidized), L-glutamic acid, (S)-glutamic acid and thromboxane B2 (TXB2), which are involved in glutamatergic synapse metabolism and GABAergic synapse pathways.

### FMT from the donors to mice regulated the serotonergic synapse pathway and glutamatergic synapse pathway in the brains of the mice

**Fig. 5:**
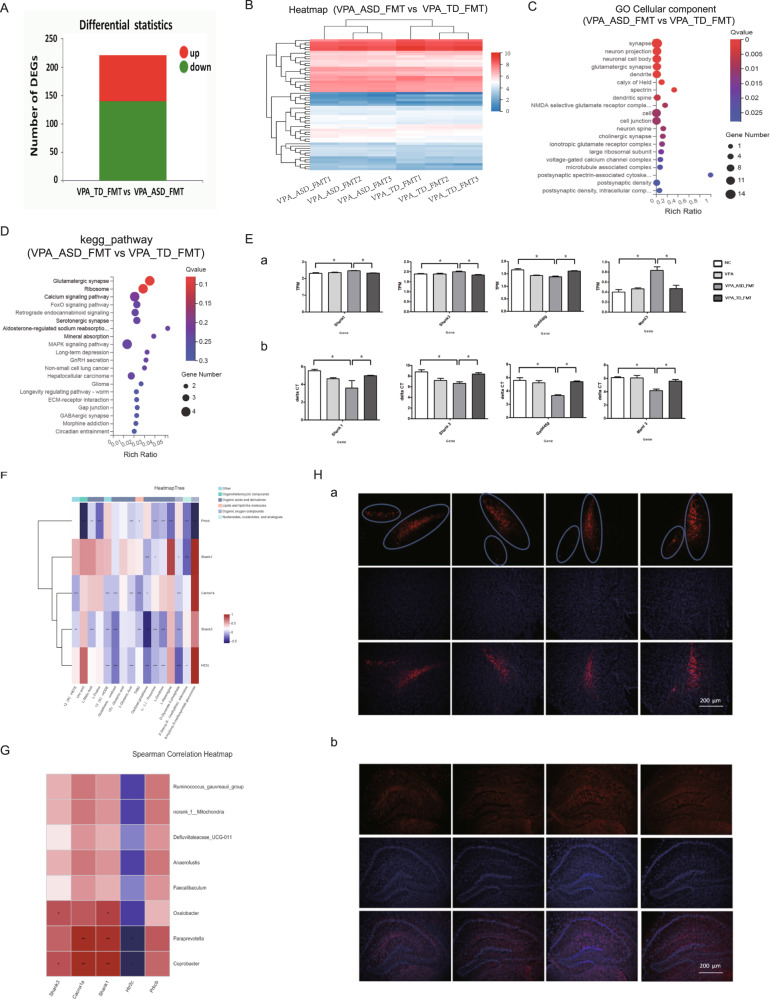
Patterns of host gene expression changes in the brain tissue of mice in response to FMT. **A**–**D** Differentially expressed genes (DEGs) involved in the brain of the VPA_TD_FMT group and the VPA_ASD_FMT group. **A** Up- and downregulation of differentially expressed genes. **B** Heatmap of DEGs in the VPA_TD_FMT group and the VPA_ASD_FMT group. Red shows upregulated DEGs, and blue shows downregulated DEGs. **C** Gene Ontology (GO) analysis of DEGs in the brain tissue of the VPA_TD_FMT group and the VPA_ASD_FMT group. **D** Enriched KEGG pathways of DEGs in the brain tissue of the VPA_TD_FMT group and the VPA_ASD_FMT group. **E** Differentially expressed genes (DEGs) related to the serotonergic synapse pathway and glutamatergic synapse pathway. (**a**), (**c**) Expression of eight DEGs. (**b**), (**d**) Relative gene expression verification of eight DEGs. **F** Correlation between the changes in serum metabolites and DEGs involved in the serotonergic synapse pathway and glutamatergic synapse pathway in the brains of the VPA_TD_FMT group and the VPA_ASD_FMT group. **G** Correlation between the changes in the relative abundance of individual genera and DEGs involved in the serotonergic synapse pathway and glutamatergic synapse pathway in the brains of the VPA_TD_FMT group and the VPA_ASD_FMT group. *P*-values were corrected for multiple testing using the Benjamini‒Hochberg false discovery rate. *p < 0.05, **p < 0.01; **H** (**a**) Tyrosine hydroxylase (TH) immunofluorescence staining of the mice after FMT. Microphotographs showing the distribution of dopaminergic neurons in the hippocampal region of the brain in response to FMT; (**b**) c-Fos activation patterns in the hippocampal region in response to FMT, Scale bar is 200 μm.

To understand the interactions between the gut microbiota and gene expression changes in the brain that may be involved in ASD, we employed brain RNA sequencing from the VPA_TD_FMT and VPA_ASD_FMT groups to distinguish DEGs in the brain after FMT. DEGs containing 81 upregulated genes and 141 downregulated genes were identified in the VPA_TD_FMT group versus the VPA_ASD_FMT group (Fig. 5A, B). GO cellular component analysis indicated that most of the genes that were dysregulated in the VPA_ASD_FMT group were involved in synapse, neuron projection, glutamatergic synapse, dendrite, spectrin, dendritic spine and neuron spine (Fig. 5C). KEGG pathway enrichment analysis indicated that dysregulated genes were grouped into categories to determine the regulation of the following pathways: calcium signaling pathway, MAPK signaling pathway, and important neurotransmitter systems, including glutamatergic synapse, serotonergic synapse, and GABAergic synapse (Fig. 5D). Interestingly, we found that in brain tissue, the expression of the genes Cacna1a, Prkcb and Htr2c, which are enriched in the serotonergic synapse pathway, and Cacna1a, Prkcb, Shank1 and Shank3, which are enriched in the glutamatergic synapse pathway, changed after FMT. The expression of these DEGs related to the serotonergic synapse pathway and glutamatergic synapse pathway was tested by RT‒PCR (Fig. 5E a, b).

According to the aforementioned findings, we attempted to identify the potential signaling pathways mediated by the metabolites in the brain after FMT. We performed correlation analysis between metabolic activity acquisition and gene expression in the brain (Fig. 5F, G). Links between altered brain gene expression and serum metabolites were found after FMT exposure in the VPA_TD_FMT group and the VPA_ASD_FMT group. An association between the differentially abundant metabolites and gene expression was observed. Immunofluorescence staining indicated that the expression of the gene encoding nigrostriatal tyrosine hydroxylase (TH), a rate-limiting enzyme of dopamine biosynthesis, increased after FMT from the TD, and C-fos, a neural activation marker, had a similar expression pattern (Fig. 5H). These results revealed that microbiota-derived signaling compounds and metabolites have a potential impact on the transcriptomic landscape of the brain, which is helpful for explaining the development and treatment of ASD.

## Discussion

ASD is a complex behavioral disorder diagnosed by social interaction difficulties, restricted verbal communication, and repetitive behaviors [22]. Although ASD etiology remains poorly understood, an increasing number of reports indicate that ASD patients have a different gut microbiota composition than healthy controls [8, 23]. Studies have shown correlations between gastrointestinal abnormalities and neuropsychiatric disorders [24]. The gut microbiome and its influence on host behavior, intestinal barrier and immune function are considered to be critical aspects of the brain-gut axis, which may contribute to a variety of neuropsychiatric disorders, including depression, schizophrenia, Parkinson’s disease, and autism [25]. An increasing number of studies have shown a link between alterations in the composition of the gut microbiota and both gastrointestinal and neurobehavioral symptoms of ASD children. Some studies have yielded inconsistent results directly linking the gut microbiome as the cause of certain behaviors such as anxiety, depression and autistic behavior. Research by Gratten and his team shows that autism-related traits and preferences are linked to food preferences, while a less diverse diet could lead to a less diverse gut microbiome and diarrhea-like stool [4]. Even though the authors could not identify a direct association between the gut microbiome and ASD, the question of whether these microbial differences drive the development of ASD still needs to be answered.

This study focused on analyzing the interactions between the gut microbiota and the host during ASD development and treatment. We transplanted fecal microbiota from autistic donors into VPA mice and compared the mouse behavior with mice transplanted using fecal microbiota from screened healthy donors. Mice with the ASD microbiome, characterized by decreased alpha- and beta-diversity, exhibited enhanced ASD-like behaviors, while mice with the healthy donor microbiome, characterized by increased alpha- and beta-diversity and a changed gut microbial structure, exhibited improved ASD-like behaviors, and FMT from ASD patients to normal mice have similar results (Supplymentary Fig. 1). Evidence has shown that the altered gut microbiota of ASD patients can influence the immune system and cause metabolite secretion [26]. The Firmicutes to Bacteroidetes (F/B) ratio is significant owing to its correlations with conditions such as obesity, microbiota maturation, dysbiosis, and systemic inflammation [27]. The most discernible difference in the F/B ratio is that it is greater in humans than in mice [28]. We found that VPA-treated ASD mice showed a higher F/B ratio than control mice. After FMT, the recipient mice receiving the transplanted microbiota have a microbiota structure similar to that of the donor, which has a potential impact on autistic behaviors. We also found two specific donor genera (*Bacteroides* and *Odoribacter*) in the gut microbiota of recipients. ASD behavioral symptoms may derive from gut microbiota dysbiosis, and FMT using healthy donors may effectively rebalance the gut microbiota and alleviate some ASD symptoms.

Physiological, cognitive, and behavioral differences in ASD patients are hypothesized to be the result of changes in gene regulation [29]. A likely mechanism is that the gut microbiomes could impact host gene regulation by regulating the expression of host genes [30, 31]. Therefore, some genes with a sensitive response to microbiome variation may play a role in ASD-related traits. We found that metabolic pathway (mainly enriched in lipid and amino acid metabolism pathways), glycosphingolipid biosynthesis, alpha-linolenic acid metabolism, and serotonergic synapse metabolism were involved in gene regulation in the colon after FMT. Disturbances in these pathways have been shown to be relevant to neurodevelopment.

The gut microbiota is a crucial regulator of host metabolism [32, 33]. By performing untargeted metabolomics analyses, we observed that the levels of several neutral amino acids, including L-glutamic acid/(S)-glutamic acid, glutathione, oxidized L-proline, and L-asparagine, changed in the serum after FMT. In the transcriptome analysis of colon biopsies, FMT also led to gene expression changes in the serotonergic synapse pathway. Interestingly, differentially expressed metabolites were enriched in the glutamatergic synapse and serotonergic synapse pathways, including L-glutamic acid/(S)-glutamic acid, glutathione, oxidized L-proline, L-asparagine and TXB2. Our findings indicated that the decrease in the abundances of the genera *Bacteroides* and *Odoribacter* resulted in gene expression changes in colon tissue and hence metabolite variation in the serum, subsequently regulating the glutamatergic system and serotonergic system in the brain. These results suggest that the microbiota-gut-brain axis plays an important role in the development and treatment of ASD. Our study found that the fecal microbiome from the donors modulated microbial community structures and improved ASD-like behaviors in VPA-induced autism mice. The regulation of glutamine metabolism and recycling pathways was associated with a decrease in the genera *Bacteroides* and *Odoribacter* after FMT. The serotonergic synapse pathway- and glutamatergic synapse pathway-related gene expression profiles were regulated, which may be the molecular basis for the changes in glutathione metabolism. In particular, the increases in the abundances of *Turicibacter* and *Alistipes* were also positively correlated with the improvement in behavior after FMT, which is in accordance with our metagenomic analysis of our ongoing clinical research project on ASD treatment with FMT. Treatment strategies targeting the gut microbiome by FMT have the potential to improve behavioral deficits associated with ASD.

This study provides valuable clues to study the relationship between the gut microbiota and ASD development and treatment. Future studies with more rigorous experimental designs are needed to validate these findings in clinical studies, the effects of certain microorganisms and metabolism on ASD, and the underlying regulatory mechanisms of FMT.

## Supplementary information

Supplementary Figure Legends

Supplymentary Figure.1

Supplementary table 1
