# Supplementary material for: Fecal microbiota transplantation improves VPA-induced ASD mice by modulating the serotonergic and glutamatergic synapse signaling pathways
Source: Transl Psychiatry. 2023 Jan 21;13:17. doi: 10.1038/s41398-023-02307-7 (PMC9859809; doi:10.1038/s41398-023-02307-7)
Supplement: Supplementary file 1 — Supplementary Figure Legends [file 41398_2023_2307_MOESM1_ESM.docx]

**Supplementary Figure legends**

Supplementary Fig. 1 **(A)** Three-chamber sociability test: After a 10-min habituation to a three-chambered box, an empty cup and a cup containing stranger 1 were introduced in the side chambers for a 10-min sociability session. Thereafter, stranger 2 was added under the empty cup for a 10-min social novelty session. **(a)** Time spent in each side chamber containing the strange 1 mouse or empty wire cage. **(b)** Time spent in each side chamber containing a familiar mouse (strange 1) or a strange 2 mouse 10 min after the first exposure. **(B)** Open-field test: **(a)** Comparison of times spent in the center zone among groups. **(b)** Total distance traveled in the entire field over 10 min of groups.. **(C)** Elevated plus maze test: **(a)** Percentages of time spent in the open arms (%) were measured. **(b)** Percentages of open arm entries (%) were measured. *, P, 0.05; ASD_FMT: mice transplanted with the fecal microflora of donors with ASD
