# Supplementary figures and images for: Fecal microbiota transplantation improves VPA-induced ASD mice by modulating the serotonergic and glutamatergic synapse signaling pathways
Source: Transl Psychiatry. 2023 Jan 21;13:17. doi: 10.1038/s41398-023-02307-7 (PMC9859809; doi:10.1038/s41398-023-02307-7)

A

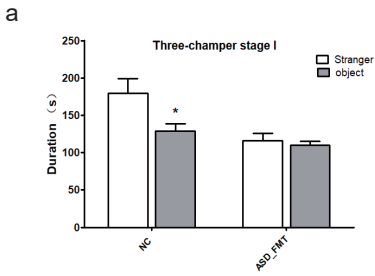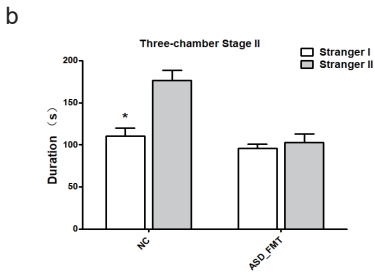

B

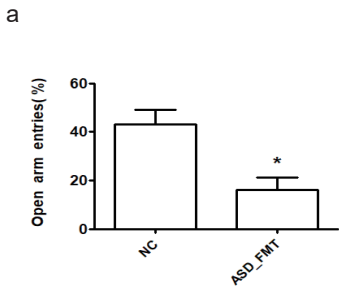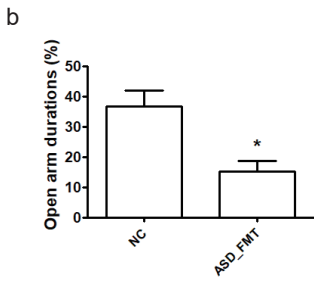

C

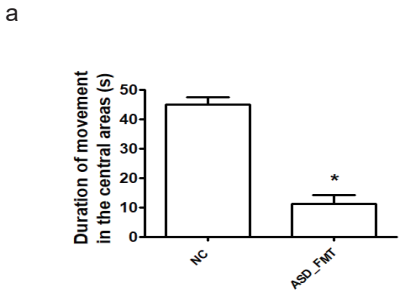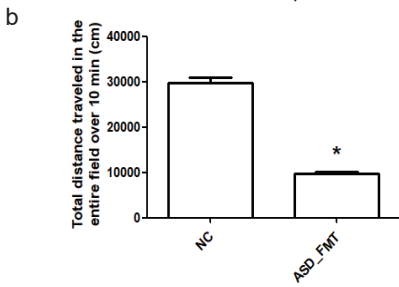

Supplement: Supplementary file 2 — Supplymentary Figure.1 [file 41398_2023_2307_MOESM2_ESM.pdf]
