# Supplementary material for: Fecal microbiota transplantation improves VPA-induced ASD mice by modulating the serotonergic and glutamatergic synapse signaling pathways
Source: Transl Psychiatry. 2023 Jan 21;13:17. doi: 10.1038/s41398-023-02307-7 (PMC9859809; doi:10.1038/s41398-023-02307-7)
Supplement: Supplementary file 3 — Supplementary table 1 [file 41398_2023_2307_MOESM3_ESM.docx]

| **Supplementary table 1.**  **Characteristics of ASD donors.** | |
| --- | --- |
| Characteristics | Total (n=4) |
| Age (mean ± SD) | 5.5±1.4 |
| Gender |  |
| Male | 3 |
| Female | 1 |
| Scales |  |
| ABC (mean ± SD) | 73.1±23.5 |
| CARS (mean ± SD) | 33.5±3.2 |

ABC, Autism Behavior Checklist;

CARS, Childhood Autism Rating Scale;
